# High expression of Solute Carrier Family 1, member 5 (SLC1A5) is associated with poor prognosis in clear-cell renal cell carcinoma

**DOI:** 10.1038/srep16954

**Published:** 2015-11-24

**Authors:** Yidong Liu, Liu Yang, Huimin An, Yuan Chang, Weijuan Zhang, Yu Zhu, Le Xu, Jiejie Xu

**Affiliations:** 1 Department of Biochemistry and Molecular Biology, School of Basic Medical Sciences, Fudan University, Shanghai, China; 2 Department of Urology, Shanghai Cancer Center, Fudan University, Shanghai, China; 3 Department of Urology, Zhongshan Hospital, Fudan University, Shanghai, China; 4 Department of Immunology, School of Basic Medical Sciences, Fudan University, Shanghai, China; 5 Department of Urology, Ruijin Hospital, School of Medicine, Shanghai Jiaotong University, Shanghai, China

## Abstract

Solute Carrier Family 1, member 5 (SLC1A5), also named as ASCT2, a major glutamine transporter, is highly expressed in various malignancies and plays a critical role in the transformation, growth and survival of cancer cells. The aim of this study was to assess the clinical significance of SLC1A5 in patients with clear-cell renal cell carcinoma (ccRCC). SLC1A5 expression was evaluated by immunohistochemistry on tissue microarrays. Kaplan-Meier method was conducted to compare survival curves. Univariate and multivariate Cox regression models were applied to assess the impact of prognostic factors on overall survival (OS). A nomogram was then constructed on the basis of the independent prognosticators identified on multivariate analysis. The predictive ability of the models was compared using Receiver operating characteristic (ROC) analysis. Our data indicated that high expression of SLC1A5 was significantly associated with advanced TNM stage, higher Fuhrman grade and shorter OS in ccRCC patients. Multivariate analysis confirmed that SLC1A5 was an independent prognosticator for OS. A nomogram integrating SLC1A5 and other independent prognosticators was constructed, which showed a better prognostic value for OS than TNM staging system. In conclusion, high SLC1A5 expression is an independent predictor of adverse clinical outcome in ccRCC patients after surgery.

Renal cell carcinoma (RCC) represents 2–3% of all adult cancers. Annually, it afflicts approximately 209,000 people and causes nearly 102,0 00 deaths around the world^1^. The incidence rate of RCC has slightly increased during the last 3 decades, contributing to a steadily increasing mortality rate worldwide despite the continuously improved clinical diagnosis and treatment^2^. Hence, it is urgent to develop early diagnostic markers, efficient therapeutic strategies and accurate prognostic factors for patients with RCC. Clear-cell RCC (ccRCC), the most common type of this disease, has been characterized as a metabolic disorder. Oncogenic metabolism has been presented as a critical feature of ccRCC^3^. Thus focusing on the fundamental metabolic dysregulation in ccRCC might provide new opportunities to identify potential therapeutic targets and prognostic factors for the disease.

Although glutamine is a nonessential amino acid which can be synthesized from glucose, several cancers indulge in glutamine consumption, even cannot survive without exogenous glutamine. Glutamine metabolism plays a central role in tumor development^4^. Glutamine performs as a nitrogen donor for nucleotide and protein synthesis. After donating its amide group to participate both purine and pyrimidine synthesis, glutamine is converted to glutamate, which is the main nitrogen donor for the synthesis of nonessential amino acids^5^. Also, glutamate is the precursor of the major cellular antioxidant, glutathione^6^. Glutamate can be exploited to form α-ketoglutarate, an important tricarboxylic acid (TCA) cycle metabolite, when losing its amine group. Then, α-ketoglutarate can be converted to citrate via several enzymatic steps, which would both produce acetyl-coA for the lipid synthesis and be converted to malate for the subsequent production of NADPH^4^. Furthermore, glutamine has been regarded as an essential activator for the mammalian target of rapamycin complex (mTORC)1, which regulates protein translation, cell growth and macroautophagy^4^,^7^. Besides being utilized in anabolic metabolism, the exported glutamine serves as stimuli for the uptake of essential amino acids, which directly activates mTORC1 and protein synthesis^8^. Consequently, glutamine plays a pivotal role in many cancers including ccRCC. Cross-platform molecular analyses have revealed that increased glutamine transport expression correlated with poor prognosis in patients with ccRCC^9^.

Glutamine can be transported by four families of amino acid transporter systems including sodium-independent system L and system b^0,+^ and sodium-dependent system A, B^0,+^, y + L, ASC and N. Among them, system ASC is the most ubiquitously expressed one in human cancer cells^10^. Solute Carrier Family 1, member 5 (SLC1A5), also named as ASCT2, belongs to system ASC and performs as a high-affinity glutamine transporter in cancer cells^11^. Consistent with the functions exerted by glutamine, SLC1A5 is profoundly involved in uptake of essential amino acids, activation of mTORC1 and glutamine-dependent tumor cell survival and growth^8^. To date, SLC1A5 has been regarded as an indispensable “switch” of glutamine metabolism and thus to be a critical regulator for cancer development^12^. Several studies have revealed that SLC1A5 is highly expressed in several cancer types and its expression is closely correlated with tumor development and prognosis^13^,^14^,^15^,^16^,^17^,^18^. It also has been determined that the mRNA level of SLC1A5 is significantly higher in tumor tissues than normal tissues of patients with ccRCC^9^. However, the protein level and clinical significance of SLC1A5 expression in ccRCC remains unclear.

Here, we sought to investigate the association of SLC1A5 expression with clinicopathologic features and patient outcomes. Moreover, a nomogram integrating SLC1A5 expression and pathologic variables was established to help predict prognosis and guide management for ccRCC patients after surgery.

## Results

### Immunohistochemical finding and association between SLC1A5 expression and clinicopathologic characteristics in patients with ccRCC

**Figure 1:**
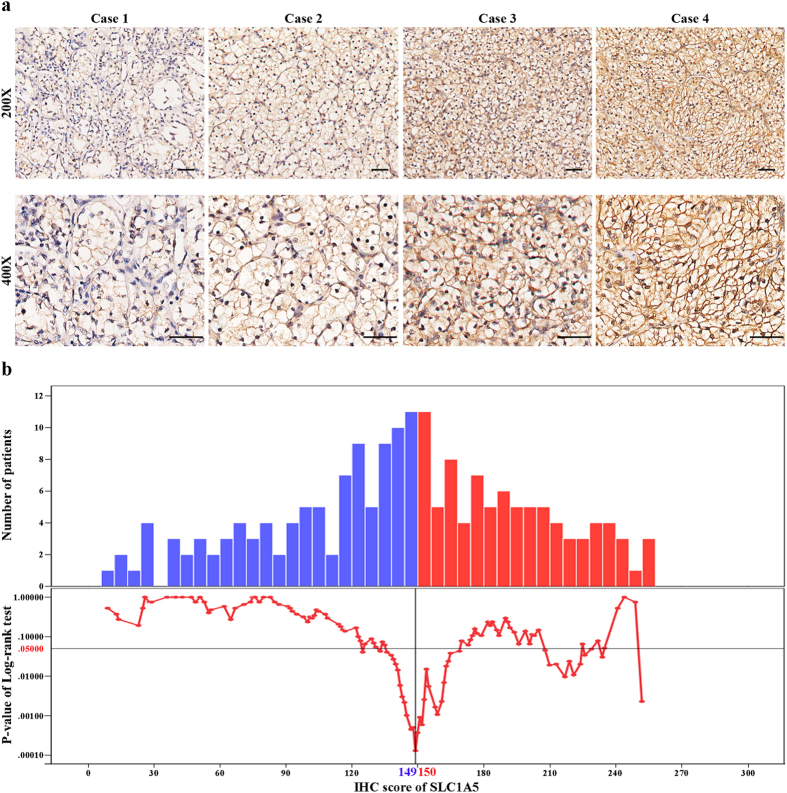
SLC1A5 expression in clear-cell renal cell carcinoma (ccRCC) tissues and dichotomization of patients based on the cutoff of H-score. (**a**) Representative immunohistochemical (IHC) images of ccRCC tissues with differential expression level of SLC1A5. Case 1 (H-score) = 0 × 69 (%) + 1 × 26 (%) + 2 × 5 (%) + 3 × 0 (%) = 36; Case 2 (H-score) = 0 × 22 (%) + 1 × 51 (%) + 2 × 27 (%) + 3 × 0 (%) = 105; Case 3 (H-score) = 0 × 8 (%) + 1 × 40 (%) + 2 × 32 (%) + 3 × 20 (%) = 164; Case 4 (H-score) = 0 × 0 (%) + 1 × 5 (%) + 2 × 41 (%) + 3 × 54 (%) = 249. Scale bar: 50 μm (original magnification × 200, × 400). (**b**) Dichotomization of patients based on the cutoff of H-score selected via “minimum P value” approach.

To investigate whether the expression pattern of SLC1A5 is altered during tumorigenesis of ccRCC, we evaluated SLC1A5 expression levels in normal kidney tissues (n = 3), ccRCC tumor tissues (n = 10) and corresponding peri-tumor tissues (n = 10) by qRT-PCR analysis. As presented in Figure S1, SLC1A5 expression was significantly upregulated in tumor tissues when compared with corresponding peri-tumor tissues (*P* = 0.003) and normal kidney tissues (*P* = 0.007). To further evaluate the protein level of SLC1A5 in ccRCC tumor tissues, we detected the expression of SLC1A5 by immunohistochemical staining analysis in 187 patients with ccRCC. As presented in Fig. 1a, the expression of SLC1A5 was mainly appeared in the membrane of tumor cells and the staining intensity and distribution were variable in different specimens. The H-score of SLC1A5 expression ranged from 9 to 255, According to the “minimum P value” approach conducted by X-tile, 149 was determined as the cutoff to dichotomize the patients into SLC1A5 low group (score, 9–149; n = 101) and SLC1A5 high group (score, 150–255; n = 86) (Fig. 1b).

As summarized in Table 1, patients with higher expression of SLC1A5 significantly tended to diagnosed with higher TNM stage (*P* = 0.032) and higher Fuhrman grade (*P* = 0.015). No other clinicopathologic variables were presented to have a significant correlation with SLC1A5 expression.

### SLC1A5 expression correlated with OS, but not RFS, of patients with ccRCC

**Figure 2:**
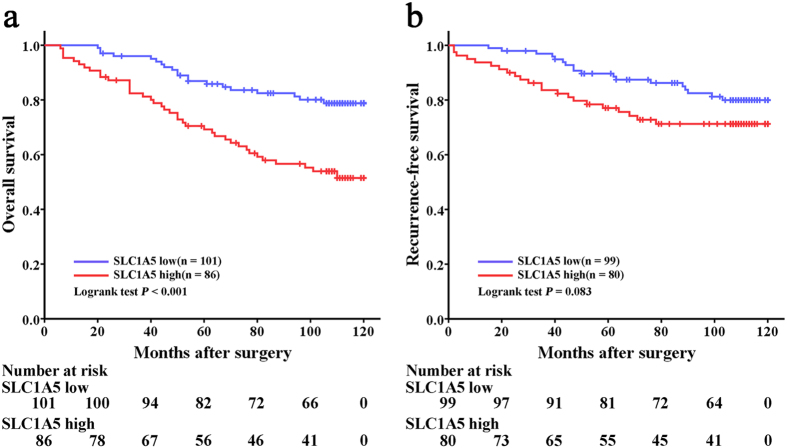
Overall survival (OS) and Recurrence-free survival (RFS) analysis of patients with ccRCC based on dichotomized SLC1A5 expression. (**a**) Kaplan-Meier analysis of OS (n = 187). (**b**) Kaplan-Meier analysis of RFS (n = 179). Eight patients with tumor metastasis at the time of surgical operation were excluded from the RFS analysis as indicated by the end point of RFS. *P* value was calculated by log-rank test.

Kaplan-Meier method and log-rank test were performed to assess the relationship between SLC1A5 expression and clinical outcome of patients with ccRCC. The median follow-up was 106 months (range, 6–120 months). As shown in Fig. 2a, SLC1A5 expression was significantly associated with OS (*P* < 0.001) of ccRCC patients, and so that means higher SLC1A5 expression indicated earlier death. Nevertheless, SLC1A5 expression presented no significant correlation with RFS of patients (*P* = 0.083, Fig. 2b).

In order to further confirm the findings, we split the patients into quartiles according to the H-score (subgroup1: 9–103; subgroup2: 104–145; subgroup3: 146–184; subgroup4: 185–255) and re-performed survival analysis. As presented in Figure S2, patients could be significantly stratified by quadrifid SLC1A5 expression for overall survival analysis (Figure S2a, *P* = 0.005), whereas the recurrence-free survival analysis still cannot reach significant level (Figure S2b, *P* = 0.097).

### SLC1A5 expression was identified as an independent prognosticator in patients with ccRCC

Univariate cox analysis was conducted to evaluate the prognostic significance of clinicopathologic variables in ccRCC. As presented in Table 2, tumor size (*P* < 0.001), pT-stage (*P* < 0.001), pN-stage (*P* < 0.001), metastasis (*P* < 0.001), Fuhrman grade (*P* < 0.001), microvascular invasion (*P* = 0.004), necrosis (*P* < 0.001), sarcomatoid (*P* < 0.001), ECOG-PS (Eastern Cooperative Oncology Group performance status) (*P* = 0.001) and SLC1A5 (*P* < 0.001) were all have a significant impact on OS.

Then the above significant factors were brought into the multivariate cox analysis. Results in Table 2 indicated that pT-stage (*P* = 0.005), pN-stage (*P* = 0.043), metastasis (*P* < 0.001), Fuhrman grade (*P* = 0.048), sarcomatoid (*P* = 0.038) and SLC1A5 (*P* = 0.001) in multivariate analysis still had statistical significance and were determined as independent prognostic factors in ccRCC.

### Prognostic nomogram for survival of patients with ccRCC

**Figure 3:**
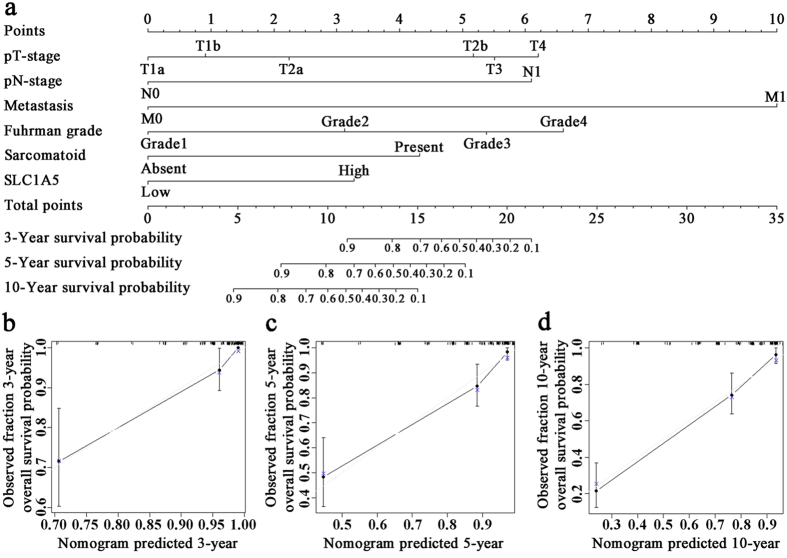
Nomogram and calibration plot for prediction of OS in patients with ccRCC. Postoperative prognostic nomogram of patients with ccRCC (**a**). The calibration plots for predicting survival at 3 years (**b**), 5 years (**c**) and 10 years (**d**).

Further multivariate analysis was conducted among the independent prognostic factors (Table 2). Thus we constructed a prognostic nomogram via integrating all these independent prognostic indicators for OS (Fig. 3a). The calibration plot for the probability of overall-survival at 3-, 5- or 10-year after surgery presented an optimal agreement between actual observation and the prediction by nomogram (Fig. 3b-d).

### Superior performance of the nomogram-based model

According to the nomogram, each subgroup of every independent prognostic indicator had been assigned a risk score (Table 3). Then each patient in this study was scored by the nomogram based risk score.

**Figure 4:**
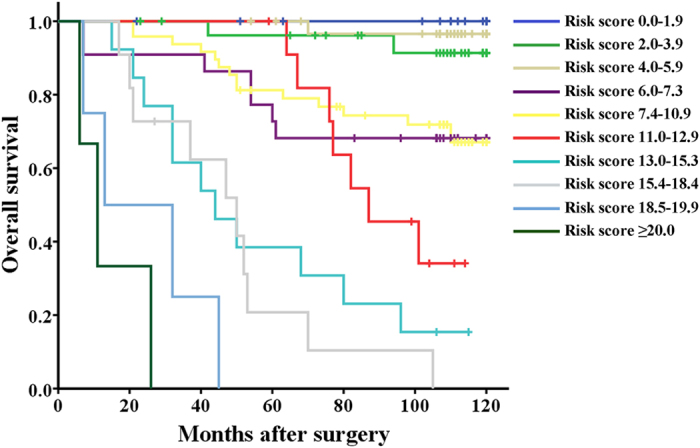
Kaplan–Meier curves of overall survival based on risk score calculated by nomogram. Statistical differences between each two subgroups were assessed by log-rank test.

**Figure 5:**
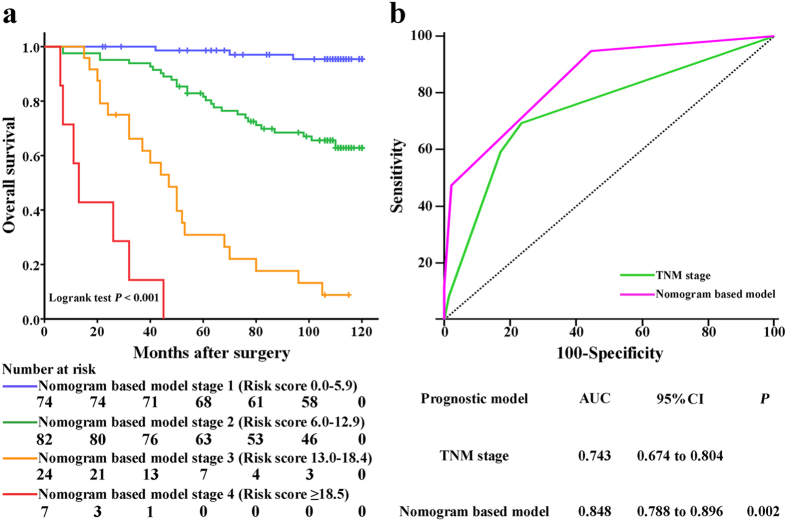
Kaplan-Meier analysis and receiver operating characteristic (ROC) analysis of nomogram-based model. (**a**) Kaplan-Meier analysis of patients according to stages of nomogram-based model. P value was calculated by log-rank test. (**b**) ROC analysis of the sensitivity and specificity for the prediction of OS of TNM staging system and nomogram-based model.

Patients were preliminarily stratified according to the nomogram-based risk scores by Kaplan-Meier curves (Fig. 4) and eventually grouped into four subgroups via combining the curves with similar prognosis. The newly established nomogram-based model included four risk subgroups which represented significant distinct prognosis with each other (Fig. 5a, *P* < 0.001).

To further determine the effectiveness of the nomogram-based model, receiver operating characteristic analysis was conducted. As presented in Fig. 5b, the nomogram-based model (Area under curve [AUC], 0.848; 95% confidence interval [CI], 0.788–0.896) showed significantly higher prognostic accuracy than TNM staging system (AUC, 0.743; 95%CI, 0.674–0.804, *P* = 0.002).

## Discussion

To our knowledge, this study was the first to report an association between high SLC1A5 expression and poor prognosis in ccRCC patients following surgery. Moreover, SLC1A5 expression has been identified as an independent prognostic factor and could be used to construct a nomogram with established pathologic factors. This work indicated that SLC1A5 might play an important role in the development of ccRCC. Recent studies demonstrated that SLC1A5, the primary glutamine transporter, could promote tumor cell growth, cell cycle progression and survival in neuroblastoma, colorectal cancer, breast cancer and prostate cancer^19^,^20^,^21^,^22^. As glutamine is an important immunomodulatory nutrient, SLC1A5 also has been determined to involve in inflammatory T cell responses, which might exert key functions in tumor immunity^23^. Thus, the molecular mechanism and functional significance of SLC1A5 in ccRCC merits further investigation.

SLC1A5 expression has been detected in brain, lung, skeletal muscle, testis, adipose tissue, large intestine and kidney^24^. As an important neutral amino acid transporter, SLC1A5 has been documented to transport serine, alanine, cysteine, threonine, glutamine, asparagine and so on^24^. Its ubiquitous tissue expression, along with its ability to transport certain key amino acids, indicates that SLC1A5 plays a crucial role in physiological processes including glutamine homeostasis, embryogenesis and retroviral infection^12^,^25^,^26^. In the current study, our initial work revealed that SLC1A5 expression was markedly upregulated in tumor tissues when compared with corresponding peri-tumor tissues and normal kidney tissues, which drawn our attention on the clinical significance of SLC1A5 expression in tumor tissues of ccRCC patients.

Consistent with previous findings in other cancers types, high SLC1A5 expression was identified as an independent adverse prognostic factor for OS in ccRCC. Considering the heterogeneity of ccRCC and its unpredictable natural history, SLC1A5 might be useful in risk stratification and personalizing postsurgical surveillance. Furthermore, the nomogram constructed in our study performed well in outcome prediction and provided a novel prognostic system for clinical practice.

Besides its significance in prognosis, the value of SLC1A5 in cancer treatment has also drawn more and more attention these years. Indeed, studies have demonstrated that blocking glutamine uptake might be an attractive strategy for cancer therapy and SLC1A5 represents as a potential therapeutic target in this pathway^27^,^28^. Since it is profoundly involved in mTOR activation, SLC1A5 exerts functions partly via mTOR signaling and thus inhibiting SLC1A5 expression could diminish the oncogenic effect of mTOR pathway in several cancer types^29^,^30^,^31^. mTOR signaling has been revealed to be maladjusted in ccRCC and molecularly targeted therapies against mTOR have gradually become one of the mainstream strategies in treatment of advanced ccRCC^32^. Hence, SLC1A5 might be an appealing target in the treatment of ccRCC, which also needs our further exploration.

Although the clinical significance of SLC1A5 in ccRCC has been revealed, several limitations of this study warrant further discussion. Firstly, the number of patients enrolled in this study was small, especially for the patients with advanced disease. Secondly, an independent external cohort is necessary to confirm our findings. Thirdly, the association between SLC1A5 expression and mTOR activation in ccRCC needs to be demonstrated. Finally, functional studies should be conducted to uncover the biological mechanisms of SLC1A5 in ccRCC.

In summary, the current study demonstrated that high SLC1A5 expression was an independent adverse prognostic factor for OS in ccRCC patients. A prognostic nomogram integrating SLC1A5 expression and pathologic factors may improve the postsurgical management of ccRCC patients. Functional studies are needed to evaluate the molecular mechanisms of SLC1A5 in the tumorigenesis of ccRCC and its role as a therapeutic target.

## Methods

### Patients

All methods were approved by the research medical ethics committee of Fudan University and were carried out in accordance with the approved guidelines. Informed consent on the use of clinical specimens had been received from each patient. A total of 187 consecutive patients with ccRCC who underwent radical or partial nephrectomy at Zhongshan Hospital, Fudan University (Shanghai, China) were enrolled to construct the tissue microarray in the current study. These specimens of patients were collected between January 2003 and December 2004. Another three normal kidney tissues and ten pairs of tumor tissues with corresponding peri-tumor tissues were obtained to isolate RNAs from the same institution. Patients who received preoperative neoadjuvant and/or postoperative adjuvant therapy were excluded in this study. Tumor stages were histologically classified according to 2010 AJCC TNM classification^33^. Overall survival (OS) and recurrence-free survival (RFS) were calculated from the date of surgery to the date of death (or the last follow-up) or to the date of recurrence (or the last follow-up), respectively. Patients with tumor metastasis at the time of surgical operation were excluded from the RFS analysis as indicated by the end point of RFS.

#### RNA extraction and quantitative real-time PCR (qRT-PCR)

Total RNA was isolated from clinical samples using TRizol reagent (Invitrogen, Carlsbad, CA) according to the manufacturer’s protocol. qRT-PCR analysis was performed as described in our previous study^34^. *GAPDH* was used as internal control. Three independent experiments were performed and each sample was detected in triplicate. *SLC1A5* forward primer: 5′-GACCGTACGGAGTCGAGAAG-3′, *SLC1A5* reverse primer: 5′- GGGGGTTTCCTTCCTCAGTG-3′; *GAPDH* forward primer: 5′- GTCAAGGCTGAGAACGGGAA-3′, *GAPDH* reverse primer: 5′- AAATGAGCCCCAGCCTTCTC-3′.

### Immunohistochemistry

Tissue microarrays and immunohistochemistry analysis were performed as previously described^35^. Primary anti-SLC1A5 antibody (1:300; Abcam, Cambridge, MA) was used for immunohistochemistry staining. The intensity of immunostaining was evaluated by two independent pathologists without the knowledge of clinicopathological data. A semi-quantitative H-score which ranged from 0 to 300 was calculated by multiplying the staining intensities (0: negative, 1: weak, 2: moderate, 3: strong) by the distribution areas (percentage of positive staining cancer cells, 0–100%) at each intensity level for each sample.

### Statistical Analysis

X-tile plot analysis was conducted to select the optimum cutoff of the H-score to dichotomize the patients into low and high groups^36^. Comparisons between SLC1A5 expression and clinicopathologic variables were evaluated using Student’s t test, χ2–test and Wilcoxon rank-sum test, as appropriate. Survival curves were conducted by Kaplan-Meier method and compared by log-rank test. Univariate and multivariate Cox proportional-hazard models were exploited to evaluate the hazard ratios and 95% confidence intervals of clinicopathologic variables. Nomogram was set to construct the prognostic model. Calibration plot was used to evaluate the prognostic accuracy of the models. Receiver operating characteristic analysis was conducted to compare the sensitivity and specificity for the prediction of OS by the prognostic models. All statistical tests were two-tailed and differences were considered significant at level of <0.05. Data were analyzed using X-tile software v3.6.1 (Yale University, New Haven, CT, USA), IBM SPSS Statistics 21.0 (IBM Corp, Armonk, New York), MedCalc Software 11.4.2.0 (MedCalc, Mariakerke, Belgium) and R software 3.0.2 with the “rms” package (R Foundation for Statistical Computing, Vienna, Austria).

## Additional Information

**How to cite this article**: Liu, Y. *et al.* High expression of Solute Carrier Family 1, member 5 (SLC1A5) is associated with poor prognosis in clear-cell renal cell carcinoma. *Sci. Rep.* **5**, 16954; doi: 10.1038/srep16954 (2015).

## Supplementary Material

Supplementary Information

## Figures and Tables

**Table 1:** Correlation between SLC1A5 expression and patient characteristics.

Characteristic	Patients (n = 187)		SLC1A5 expression		P*
	Number	%	Low (n = 101)	High (n = 86)	
Age at surgery, years†					0.808
Mean ± SD	54.79 ± 11.67		54.59 ± 10.50	55.01 ± 12.98	
Gender					0.606
Female	59	31.55	34	25	
Male	128	68.45	67	61	
Tumor size, cm†					0.233
Mean ± SD	4.50 ± 2.63		4.29 ± 2.41	4.75 ± 2.87	
pT-stage					0.056
T1a	69	36.90	43	26	
T1b	53	28.34	26	27	
T2a	12	6.42	9	3	
T2b	3	1.60	2	1	
T3	48	25.67	21	27	
T4	2	1.07	0	2	
pN-stage					0.888
N0	184	98.40	100	84	
N1	3	1.60	1	2	
Metastasis					0.538
M0	181	96.79	99	82	
M1	6	3.21	2	4	
TNM stage					**0.032**
I	116	62.03	67	49	
II	14	7.49	11	3	
III	50	26.74	21	29	
IV	7	3.74	2	5	
Fuhrman grade					**0.015**
1	29	15.51	17	12	
2	80	42.78	52	28	
3	51	27.27	23	28	
4	27	14.44	9	18	
MVI					0.585
Absent	150	80.21	83	67	
Present	37	19.79	18	19	
Necrosis					0.140
Absent	143	76.47	82	61	
Present	44	23.53	19	25	
Sarcomatoid					0.745
Absent	172	91.98	94	78	
Present	15	8.02	7	8	
ECOG-PS					0.381
0	158	84.49	88	70	
≥1	29	15.51	13	16	

SLC1A5 = Solute Carrier Family 1, member 5; SD = standard deviation;

MVI = microvascular invasion; ECOG PS = Eastern Cooperative Oncology Group performance status.

*P < 0.05 is considered statistically significant.

^†^The results of continuous variables are presented as mean ±SD (standard deviation).

**Table 2:** Univariate and Multivariate Cox regression analysis of Overall survival.

Characteristic	Univariate analysis	Multivariate analysis		Selected Factors for Building the Model	
	P*	Hazard Ratio (95% CI)	P*	Hazard Ratio (95% CI)	P*
Age at surgery, years	0.553				
Gender	0.821				
Female					
Male					
Tumor size, cm	**<0.001**	1.053(0.936–1.184)	0.393		
pT-stage	**<0.001**		**0.005**		**<0.001**
T1a		Reference		Reference	
T1b		1.263(0.496–3.219)	0.625	1.322(0.542–3.227)	0.539
T2a		1.908(0.497–7.326)	0.347	1.979(0.525–7.457)	0.313
T2b		2.311(0.281–18.998)	0.436	4.670(0.878–24.847)	0.071
T3		4.382(1.698–11.307)	**0.002**	5.184(2.251–11.939)	**<0.001**
T4		6.116(0.425–88.114)	0.183	6.520(0.473–89.928)	0.161
pN-stage	**<0.001**		**0.043**		**0.017**
N0		Reference		Reference	
N1		5.910(1.060–32.953)		6.208(1.383–27.853)	
Metastasis	**<0.001**		**<0.001**		**<0.001**
M0		Reference		Reference	
M1		23.478(6.793–81.142)		20.086(5.947–67.835)	
Fuhrman grade	**<0.001**		**0.048**		**0.016**
1		Reference		Reference	
2		2.683(0.532–13.527)	0.232	2.583(0.524–12.727)	0.244
3		5.011(1.019–24.653)	**0.047**	5.044(1.035–24.568)	**0.045**
4		6.145(1.129–33.443)	**0.036**	7.320(1.394–38.448)	**0.019**
MVI	**0.004**		0.063		
Absent		Reference			
Present		1.855(0.967–3.562)			
Necrosis	**<0.001**		0.963		
Absent		Reference			
Present		1.017(0.505–2.045)			
Sarcomatoid	**<0.001**		**0.038**		**0.005**
Absent		Reference		Reference	
Present		2.986(1.061–8.407)		3.584(1.457–8.815)	
ECOG-PS	**0.001**		0.962		
0		Reference			
≥1		1.018(0.491–2.112)			
SLC1A5	**<0.001**		**0.001**		**0.001**
Low		Reference		Reference	
High		2.956(1.595–5.478)		2.655(1.482–4.757)	

SLC1A5 = Solute Carrier Family 1, member 5; CI = confidence interval;

MVI = microvascular invasion; ECOG PS = Eastern Cooperative Oncology Group performance status.

*P < 0.05 is considered statistically significant.

**Table 3:** Point assignment and nomogram-based risk score.

Characteristic	Nomogram-based risk score
pT-stage	
T1a	0
T1b	0.9
T2a	2.2
T2b	5.2
T3	5.5
T4	6.2
pN-stage	
N0	0
N1	6.1
Metastasis	
M0	0
M1	10
Fuhrman grade	
1	0
2	3.1
3	5.4
4	6.6
Sarcomatoid	
Absent	0
Present	4.3
SLC1A5	
Low	0
High	3.3

SLC1A5 = Solute Carrier Family 1, member 5.

## References

[b1] Gupta K., Miller J. D., Li J. Z., Russell M. W. & Charbonneau C. Epidemiologic and socioeconomic burden of metastatic renal cell carcinoma (mRCC): a literature review. Cancer Treat Rev 34, 193–205, 10.1016/j.ctrv.2007.12.001 (2008). 18313224

[b2] Lindblad P. Epidemiology of renal cell carcinoma. Scand J Surg 93, 88–96 (2004). 15285559 10.1177/145749690409300202

[b3] Gatto F., Nookaew I. & Nielsen J. Chromosome 3p loss of heterozygosity is associated with a unique metabolic network in clear cell renal carcinoma. Proc Natl Acad Sci USA 111, E866–875, 10.1073/pnas.1319196111 (2014). 24550497 PMC3948310

[b4] Wise D. R. & Thompson C. B. Glutamine addiction: a new therapeutic target in cancer. Trends Biochem Sci 35, 427–433, 10.1016/j.tibs.2010.05.003 (2010). 20570523 PMC2917518

[b5] Young V. R. & Ajami A. M. Glutamine: the emperor or his clothes? J Nutr 131, 2449S–2459S; discussion 2486S-2447S (2001). 11533293 10.1093/jn/131.9.2449S

[b6] Hensley C. T., Wasti A. T. & DeBerardinis R. J. Glutamine and cancer: cell biology, physiology, and clinical opportunities. J Clin Invest 123, 3678–3684, 10.1172/JCI69600 (2013). 23999442 PMC3754270

[b7] Wullschleger S., Loewith R. & Hall M. N. TOR signaling in growth and metabolism. Cell 124, 471–484, 10.1016/j.cell.2006.01.016 (2006). 16469695

[b8] Nicklin P. *et al.* Bidirectional transport of amino acids regulates mTOR and autophagy. Cell 136, 521–534, 10.1016/j.cell.2008.11.044 (2009). 19203585 PMC3733119

[b9] Network., C. G. A. R. Comprehensive molecular characterization of clear cell renal cell carcinoma. Nature 499, 43–49, 10.1038/nature12222 (2013). 23792563 PMC3771322

[b10] Pingitore P. *et al.* Large scale production of the active human ASCT2 (SLC1A5) transporter in Pichia pastoris–functional and kinetic asymmetry revealed in proteoliposomes. Biochim Biophys Acta 1828, 2238–2246, 10.1016/j.bbamem.2013.05.034 (2013). 23756778

[b11] Kekuda R. *et al.* Cloning of the sodium-dependent, broad-scope, neutral amino acid transporter Bo from a human placental choriocarcinoma cell line. J Biol Chem 271, 18657–18661 (1996). 8702519 10.1074/jbc.271.31.18657

[b12] Fuchs B. C. & Bode B. P. Amino acid transporters ASCT2 and LAT1 in cancer: partners in crime? Semin Cancer Biol 15, 254–266, 10.1016/j.semcancer.2005.04.005 (2005). 15916903

[b13] Witte D., Ali N., Carlson N. & Younes M. Overexpression of the neutral amino acid transporter ASCT2 in human colorectal adenocarcinoma. Anticancer Res 22, 2555–2557 (2002). 12529963

[b14] Li R. *et al.* Expression of neutral amino acid transporter ASCT2 in human prostate. Anticancer Res 23, 3413–3418 (2003). 12926082

[b15] Shimizu K. *et al.* ASC amino-acid transporter 2 (ASCT2) as a novel prognostic marker in non-small cell lung cancer. Br J Cancer 110, 2030–2039, 10.1038/bjc.2014.88 (2014). 24603303 PMC3992511

[b16] Kim S., Jung W. H. & Koo J. S. The expression of glutamine-metabolism-related proteins in breast phyllodes tumors. Tumour Biol 34, 2683–2689, 10.1007/s13277-013-0819-7 (2013). 23636801

[b17] Toyoda M. *et al.* Prognostic significance of amino-acid transporter expression (LAT1, ASCT2, and xCT) in surgically resected tongue cancer. Br J Cancer 110, 2506–2513, 10.1038/bjc.2014.178 (2014). 24762957 PMC4021522

[b18] Kaira K. *et al.* Clinicopathological significance of ASC amino acid transporter-2 expression in pancreatic ductal carcinoma. Histopathology 66, 234–243, 10.1111/his.12464 (2015). 24845232

[b19] Ren P. *et al.* ATF4 and N-Myc coordinate glutamine metabolism in MYCN-amplified neuroblastoma cells through ASCT2 activation. J Pathol 235, 90–100, 10.1002/path.4429 (2015). 25142020

[b20] Huang F. *et al.* Upregulated SLC1A5 promotes cell growth and survival in colorectal cancer. Int J Clin Exp Pathol 7, 6006–6014 (2014). 25337245 PMC4203216

[b21] Venmar K. T., Kimmel D. W., Cliffel D. E. & Fingleton B. IL4 receptor alpha mediates enhanced glucose and glutamine metabolism to support breast cancer growth. Biochim Biophys Acta 1853, 1219–1228, 10.1016/j.bbamcr.2015.02.020 (2015). 25746764 PMC4380623

[b22] Wang Q. *et al.* Targeting ASCT2-mediated glutamine uptake blocks prostate cancer growth and tumour development. J Pathol, 10.1002/path.4518 (2015). PMC4973854 25693838

[b23] Nakaya M. *et al.* Inflammatory T cell responses rely on amino acid transporter ASCT2 facilitation of glutamine uptake and mTORC1 kinase activation. Immunity 40, 692–705, 10.1016/j.immuni.2014.04.007 (2014). 24792914 PMC4074507

[b24] Kanai Y. *et al.* The SLC1 high-affinity glutamate and neutral amino acid transporter family. Mol Aspects Med 34, 108–120, 10.1016/j.mam.2013.01.001 (2013). 23506861

[b25] Adeva M. M., Souto G., Blanco N. & Donapetry C. Ammonium metabolism in humans. Metabolism 61, 1495–1511, 10.1016/j.metabol.2012.07.007 (2012). 22921946

[b26] Marin M., Lavillette D., Kelly S. M. & Kabat D. N-linked glycosylation and sequence changes in a critical negative control region of the ASCT1 and ASCT2 neutral amino acid transporters determine their retroviral receptor functions. J Virol 77, 2936–2945 (2003). 12584318 10.1128/JVI.77.5.2936-2945.2003 PMC149750

[b27] Willems L. *et al.* Inhibiting glutamine uptake represents an attractive new strategy for treating acute myeloid leukemia. Blood 122, 3521–3532, 10.1182/blood-2013-03-493163 (2013). 24014241 PMC3829119

[b28] Wang Q. *et al.* Targeting glutamine transport to suppress melanoma cell growth. Int J Cancer 135, 1060–1071, 10.1002/ijc.28749 (2014). 24531984

[b29] Fuchs B. C., Finger R. E., Onan M. C. & Bode B. P. ASCT2 silencing regulates mammalian target-of-rapamycin growth and survival signaling in human hepatoma cells. Am J Physiol Cell Physiol 293, C55–63, 10.1152/ajpcell.00330.2006 (2007). 17329400

[b30] Hassanein M. *et al.* SLC1A5 mediates glutamine transport required for lung cancer cell growth and survival. Clin Cancer Res 19, 560–570, 10.1158/1078-0432.CCR-12-2334 (2013). 23213057 PMC3697078

[b31] Jeon Y. J. *et al.* Regulation of Glutamine Carrier Proteins by RNF5 Determines Breast Cancer Response to ER Stress-Inducing Chemotherapies. Cancer cell 27, 354–369, 10.1016/j.ccell.2015.02.006 (2015). 25759021 PMC4356903

[b32] Rini B. I. *et al.* Randomized phase III trial of temsirolimus and bevacizumab versus interferon alfa and bevacizumab in metastatic renal cell carcinoma: INTORACT trial. J Clin Oncol 32, 752–759, 10.1200/JCO.2013.50.5305 (2014). 24297945

[b33] Edge S. B. & Compton C. C. The American Joint Committee on Cancer: the 7th edition of the AJCC cancer staging manual and the future of TNM. Ann Surg Oncol 17, 1471–1474, 10.1245/s10434-010-0985-4 (2010). 20180029

[b34] Xu J. *et al.* Hepatitis B virus X protein blunts senescence-like growth arrest of human hepatocellular carcinoma by reducing Notch1 cleavage. Hepatology 52, 142–154, 10.1002/hep.23613 (2010). 20578140

[b35] Xu L. *et al.* Prognostic value of diametrically polarized tumor-associated macrophages in renal cell carcinoma. Ann Surg Oncol 21, 3142–3150, 10.1245/s10434-014-3601-1 (2014). 24615178

[b36] Camp R. L., Dolled-Filhart M. & Rimm D. L. X-tile: a new bio-informatics tool for biomarker assessment and outcome-based cut-point optimization. Clin Cancer Res 10, 7252–7259, 10.1158/1078-0432.CCR-04-0713 (2004). 15534099

