# Supplementary material for: High expression of Solute Carrier Family 1, member 5 (SLC1A5) is associated with poor prognosis in clear-cell renal cell carcinoma
Source: Sci Rep. 2015 Nov 24;5:16954. doi: 10.1038/srep16954 (PMC4657035; doi:10.1038/srep16954)
Supplement: Supplementary Information [file srep16954-s1.pdf]

High expression of Solute Carrier Family 1, member 5 (SLC1A5) is associated with poor prognosis in clear-cell renal cell carcinoma

Yidong Liu, Liu Yang, Huimin An, Yuan Chang, Weijuan Zhang, Yu Zhu, Le Xu, Jiejie Xu

Supplementary information

Supplemental figure legends

Figure S1. SLC1A5 mRNA expression level in normal kidney tissues (n=3), ccRCC tumor tissues (n=10) and corresponding peri-tumor tissues (n=10). The expression levels were determined by quantitative real-time PCR analysis. *GAPDH* was used as internal control. *P* value is determined by Mann–Whitney test.

Figure S2. Overall survival (OS) and Recurrence-free survival (RFS) analysis of patients with ccRCC based on quadrifid SLC1A5 expression. (a) Kaplan-Meier analysis of OS (n=187). (b) Kaplan-Meier analysis of RFS (n=179). Patients are split into quartiles according to the H-score (subgroup1: 9-103; subgroup2: 104-145; subgroup3: 146-184; subgroup4: 185-255). Eight patients with tumor metastasis at the time of surgical operation were excluded from the RFS analysis as indicated by the end point of RFS. *P* value was calculated by log-rank test.

Figure S1

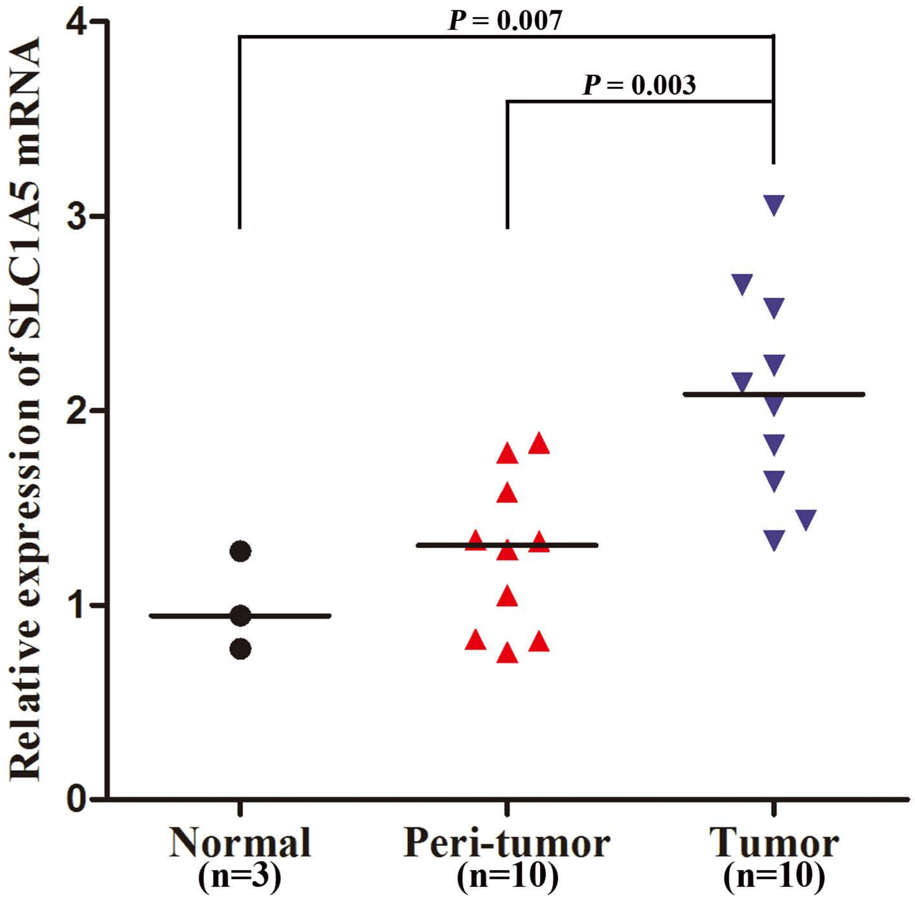

Relative expression of SLC1A5 mRNA
4
3
2
1
0
P = 0.007
P = 0.003
Normal
(n=3)
Peri-tumor
(n=10)
Tumor
(n=10)

Figure S2

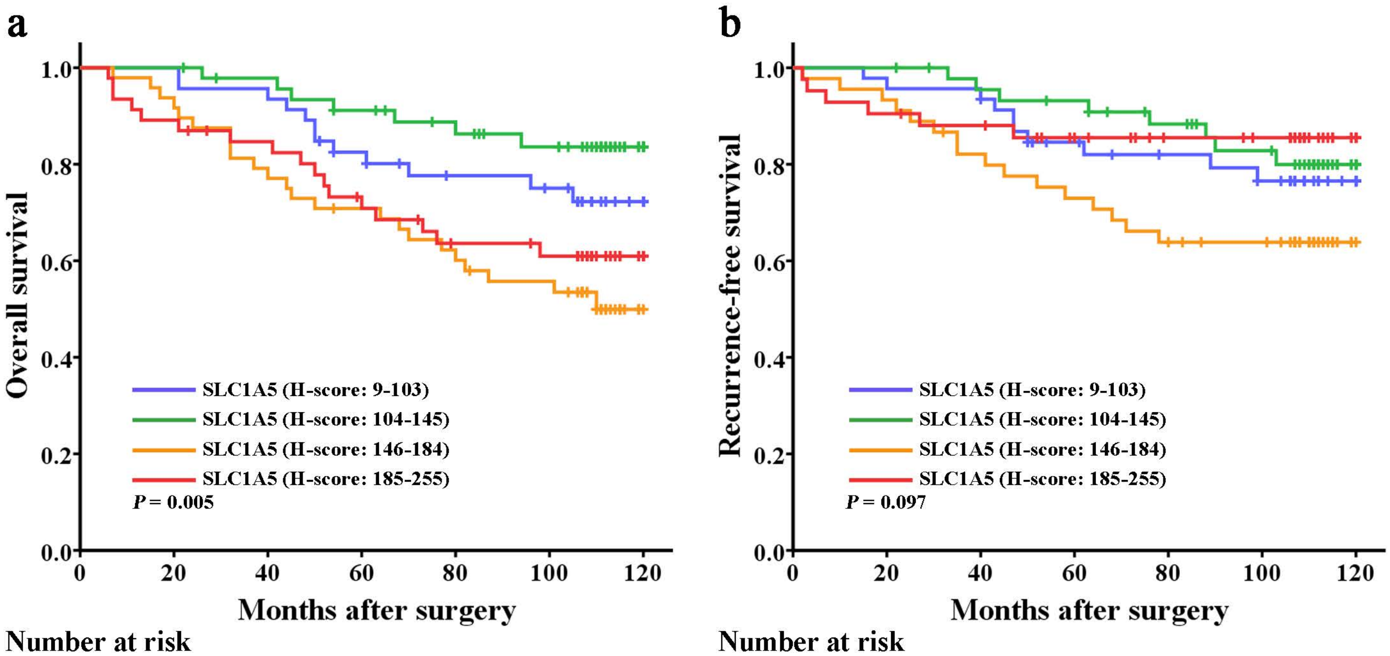

a

Number at risk

SLC1A5 (H-score: 9-103)	46	46	43	35	30	28	0
SLC1A5 (H-score: 104-145)	47	47	44	40	35	31	0
SLC1A5 (H-score: 146-184)	48	44	37	33	28	25	0
SLC1A5 (H-score: 185-255)	46	41	37	30	25	23	0

b

Number at risk

SLC1A5 (H-score: 9-103)	46	44	42	34	30	27	0
SLC1A5 (H-score: 104-145)	46	46	42	40	35	30	0
SLC1A5 (H-score: 146-184)	45	42	36	32	27	25	0
SLC1A5 (H-score: 185-255)	42	38	36	30	25	23	0
